# Association between exposure to household smoking and dental caries in preschool children: a cross-sectional study

**DOI:** 10.1186/s12199-019-0764-1

**Published:** 2019-01-26

**Authors:** Yuko Goto, Keiko Wada, Kie Konishi, Takahiro Uji, Sachi Koda, Fumi Mizuta, Michiyo Yamakawa, Kaori Watanabe, Kyoko Ando, Jun Ueyama, Takaaki Kondo, Chisato Nagata

**Affiliations:** 1 Department of Epidemiology and Preventive Medicine, Gifu University Graduate School of Medicine, 1-1 Yanagido, Gifu, 501-1194 Japan; 2 Department of Health and Welfare, Tokai Gakuin University, Gifu, Japan; 3 Department of Life and Culture, Aichi Bunkyo Women’s College, Inazawa, Aichi Japan; 4 Department of Pathophysiological Laboratory Sciences, Field of Radiological and Medical Laboratory Sciences, Nagoya University Graduate School of Medicine, Nagoya, Aichi Japan

**Keywords:** Tobacco smoke, Dental caries, Urinary cotinine, Cross-sectional studies, Preschool children

## Abstract

**Background:**

We aimed to examine the association of exposure to environmental tobacco smoke with dental caries among preschool children. Exposure to environmental tobacco smoke was assessed in terms of urinary cotinine concentrations and pack-years of exposure to smoking by parents and other family members at home.

**Methods:**

This cross-sectional study included 405 preschool children aged 3–6 years from two preschools in Japan in 2006. Information on the smoking habits of family members living with the child was obtained from parent-administered questionnaires. Dental examination was conducted to assess dental caries, that is, decayed and/or filled teeth. Urinary cotinine levels were measured using first-void morning urine samples.

**Results:**

Overall, 31.1% of the children had dental caries, and 29.5% had decayed teeth. Exposure to current maternal and paternal smoking was positively associated with the presence of dental caries after controlling for covariates. More than three pack-years of exposure to maternal smoking and more than five pack-years of exposure to smoking by all family members were significantly associated with the presence of dental caries as compared with no exposure (odds ratio [OR] = 5.55, 95% confidence interval [CI] = 2.17–14.22, *P* for trend < 0.001 and OR = 2.00, 95% CI = 1.12–3.58, *P* for trend = 0.004, respectively). These exposure variables were similarly associated with the presence of decayed teeth (OR = 2.92, 95% CI = 1.23–6.96, *P* for trend = 0.01 and OR = 1.75, 95% CI = 0.96–3.20, *P* for trend = 0.03, respectively). As compared with lowest tertile of the urinary cotinine level, the highest tertile of the urinary cotinine level was significantly associated with the presence of dental caries as well as decayed teeth; the ORs for the highest vs. lowest tertile of urinary cotinine levels were 3.10 (95% CI = 1.71–5.63, *P* for trend = 0.012) and 2.02 (95% CI = 1.10–3.70, *P* for trend = 0.10), respectively.

**Conclusions:**

These data suggest that exposure to tobacco smoke may have a dose-dependent influence on the development of caries.

## Background

It has been suggested that exposure of children to environmental tobacco smoke is associated with an increased risk of dental caries. Many studies have evaluated this association. A review of the literature by Hanioka et al. in 2011 identified 11 studies on parental or household smoking and dental caries in early childhood [1]. Significant association was reported in ten of them [2–11]. After this review, to our knowledge, six studies, including four cohort studies, have been published, and all of them showed a positive association between environmental tobacco smoke and early childhood caries [12–17]. Although data on environmental tobacco smoke and early childhood caries have been accumulated, most of these studies concerned the presence or absence of current smoking by parents or family members, and data on a dose-dependent relationship based on smoking dose have not been provided.

Cotinine is a direct metabolite of nicotine that has a high specificity for environmental tobacco smoke exposure. Cotinine concentration in saliva, blood, or urine can be an effective measure to assess a dose-dependent association of exposure to environmental tobacco smoke with dental caries [18]. To date, only one study included cotinine measurement to evaluate this association [10]. Since the cotinine level reflects short-term exposure to nicotine [18], information on long-term exposure to tobacco smoking is needed. The present study assessed the association of environmental tobacco smoke with dental caries among preschool children in terms of urinary cotinine concentrations and pack-years of exposure to smoking by parents and other family members at home.

## Methods

### Study population

This cross-sectional study included children aged 3–6 years who attended two preschools attached to Aichi Bunkyo Women’s College in Aichi Prefecture, Japan. All children at the preschools (*n* = 533) were invited for the study between October and November in 2006. Of these, 74 children were excluded because their parents declined to participate. The remaining 459 children (86.1%, 243 boys and 216 girls) were enrolled in the study. Written informed consent was obtained from parents. They completed a questionnaire asking about lifestyle factors of the children and themselves. Since 11 parents did not answer the questions about smoking status and 43 children did not attend the dental examination, the present analysis included 405 children. The questionnaire was filled in by mother (96.8%) or father (3.2%). The study protocol procedure was approved by the ethical board of the Gifu University Graduate School of Medicine, Gifu, Japan (No. 18-67 dated Sep. 6, 2006).

### Assessment of exposure to environmental tobacco smoke

We obtained information from respondents concerning the smoking habits of parents and other family members who live with the children. Respondents were asked whether a child’s mother had ever smoked. If she ever smoked, we asked at what age she started and/or quit smoking. For the smoking habits of the father and other family members, the questionnaire asked whether they had smoked since the child was born. For those who had smoked, we asked whether they had quit smoking during this time frame or were currently smoking. Likewise, considering this time frame, the smoking status of the mother, father, and other family members was classified into three categories (never, former, and current smoker). Former smokers and current smokers were asked to report years they smoked and the number of cigarettes smoked per day. Children’s pack-years of exposure to smoking at home was determined based on the smokers’ years of smoking and the number of cigarettes smoked per day since the child was born.

### Dental measurements

The School Health and Safety Act in Japan requires an execution of annual dental health check-ups for children in preschool. In accordance with the Act, a school dentist commissioned by a preschool care for and checks the oral health of children. Dental examinations were performed in a standard manner by two school dentists (one for each preschool). They detected caries lesions and tooth filling using a dental mirror under artificial light and recorded these data on dental formula. Teeth were dried and cleaned with cotton rolls or explorer if necessary. A tooth with silver fluoride varnish and fissure sealant was regarded as a filled tooth. Visible dental plaque or calculus was also recorded. Children were classified as having dental caries of deciduous teeth if they had one or more decayed or filled teeth.

### Assessment of children’s urinary cotinine

First-void morning urine samples were obtained from children with the help of their parents at their homes, although we could not obtain urine samples for six children. All urine samples were stored at − 80 °C until analysis. The total cotinine concentration (free cotinine plus its glucuronide) was measured via a solid-phase extraction (SPE) procedure and liquid chromatography–tandem mass spectrometry (LC-MS/MS) detection. The within-run precision of our method was examined through the assay of pooled urine spiked with cotinine at concentrations of 0.4, 1.7, and 10 μg/L (*n* = 4–5), and the results were less than 14.4% (relative standard deviation). The limit of detection (LOD) was 0.1 ng/ml. Urinary creatinine was measured using the conventional enzymatic method. The total urinary cotinine concentration was adjusted for creatinine (μg/mg Cr).

### Statistical analysis

Children were classified into two groups according to the presence or absence of dental caries. As preschool children (aged 3–6 years) generally have deciduous teeth, we did not analyze data separately for deciduous and permanent teeth. Children were also categorized into three levels according to pack-years of exposure to smoking by the mother, father, and other family members living with the child. The total urinary cotinine level was categorized into three levels (low, middle, or high) according to the tertile of the concentration. Sixteen children whose cotinine concentrations were below the detectable levels were assigned levels of LOD/2 (i.e., 0.05 ng/ml).

To evaluate the associations between variables for environmental tobacco smoke and the presence of dental caries, a multivariable logistic regression model was used, and the odds ratio (OR) and 95% confidence interval (CI) were calculated. The child’s sex, age, indicator for preschools, mother’s age at delivery (24 years or younger, 25–30 years, or 31 years or older), mother’s education level (12 years or less, 13–15 years, or 16 years or more), frequency of eating snacks (0, < 2, or ≥ 2 times/day), frequency of tooth brushing (0, < 2, or ≥ 2 times/day), and feeding modes until 3 months of age (breast, mixed, or bottle) were included in the models as covariates. A liner trend was assessed using continuous values for pack-years of exposure. All *P* values were calculated using a two-sided test, and a *P* value of less than 0.05 was considered statistically significant in all analyses. All statistical analyses were performed using the SAS program (Version 9.4, SAS Institute Inc., Cary NC, USA).

## Results

Characteristics of the 405 children (218 boys and 187 girls) are presented in Table 1. The mean age was 5.1. Overall, 31.1% of the children had dental caries (decayed and/or filled teeth), and 25.9% had decayed teeth. About half of the children were reported to perform tooth brushing twice or more per day. Nearly half of the children lived with current smokers at home (46.9%). Urine samples were obtained from 399 children (215 boys and 184 girls). The mean of urinary cotinine levels was 3.01 (SD 4.5) μg/mg Cr. Urine cotinine ranged from 0.03 to 39.7 μg/mg Cr. The correlation coefficient between the exposure to household smoking, i.e., the pack-years of exposure to all family smoking, and urinary cotinine level was 0.56.

**Table 1:** Basic characteristics of preschool children (*n* = 405)

Variable	*N* (%)
Child’s sex	
Boys	218 (53.8)
Girls	187 (46.2)
Age (years)	
3 ≤ 4.5	122 (30.1)
4.5 ≤ 5.5	126 (31.1)
> 5.5	157 (38.8)
Mother’s age at delivery (years)	
≤ 24	26 (6.4)
25–30	217 (53.6)
≥ 31	162 (40.0)
Maternal education level (years)	
≤ 12	127 (31.4)
13–15	197 (48.6)
≥ 16	81 (20.0)
Frequency of eating snacks (times/day)	
0	22 (5.4)
< 2	352 (86.9)
≥ 2	31 (7.7)
Frequency of tooth brushing (times/day)	
0	21 (5.2)
< 2	174 (43.0)
≥ 2	210 (51.8)
Feeding mode until 3 months old	
Breast	160 (39.5)
Mixed	178 (44.0)
Bottle	67 (16.5)
Current smoker at home (parent or household)	
Yes	190 (46.9)
No	215 (53.1)
Presence of dental caries	
Yes	126 (31.1)
No	279 (68.9)
Presence of untreated teeth	
Yes	105 (25.9)
No	300 (74.1)
Number of dental caries	
0	279 (68.9)
1	41 (10.1)
≥ 2	85 (21.0)

***N*** Number of participants

Table 2 shows the adjusted OR and 95% CI for the presence of dental caries according to environmental tobacco smoke exposure at home. Current exposure to maternal smoking was significantly associated with the presence of dental caries (OR = 3.14, 95% CI 1.56–6.31) after controlling for covariates. More than three pack-years of exposure to maternal smoking was associated with about a fivefold increased OR for dental caries as compared with no exposure. Current exposure to paternal smoking was significantly associated with the presence of dental caries, although this association was not so great as that for maternal smoking (OR = 1.64, 95% CI 1.02–2.64). More than five pack-years of exposure to smoking by all family members was significantly associated with the presence of dental caries, and the trend was significant. The total urinary cotinine level was significantly associated with the presence of dental caries after controlling for covariates (*P* for trend = 0.012).

**Table 2:** OR and 95% CI for dental caries according to environmental tobacco smoke exposure at home

	*N* with caries/total	Crude OR (95% CI)	*P* trend	Multivariate OR (95% CI)^a^	*P* trend
Exposure to maternal smoking					
Never	100/356	1.0		1.0	
Past	2/5	1.71 (0.28–10.37)		1.56 (0.21–11.48)	
Current	24/44	3.07 (1.63–5.81)		3.14 (1.56–6.31)	
Pack-years of exposure to maternal smoking					
0	100/356	1.0		1.0	
≤ 3	8/23	1.37 (0.56–3.32)		1.47 (0.56–3.84)	
> 3	18/26	5.76 (2.43–13.67)		5.55 (2.17–14.22)	
Per 1 pack-year of exposure		1.50 (1.23–1.82)	< 0.001	1.50 (1.21–1.85)	< 0.001
Exposure to paternal smoking					
Never	50/206	1.0		1.0	
Past	11/27	2.15 (0.93–4.93)		1.97 (0.81–4.76)	
Current	65/172	1.90 (1.22–2.95)		1.64 (1.02–2.64)	
Pack-years of exposure to paternal smoking					
0	50/206	1.0		1.0	
≤ 4	36/93	1.97 (1.17–3.33)		1.76 (1.00–3.10)	
> 4	40/106	1.89 (1.14–3.14)		1.63 (0.95–2.78)	
Per 1 pack-year of exposure		1.11 (1.03–1.20)	0.009	1.07 (0.99–1.17)	0.11
Pack-years of exposure to smoking by other family members					
0	111/372	1.0		1.0	
≤ 4	6/17	1.27 (0.46–3.53)		1.07 (0.35–3.25)	
> 4	9/15	3.50 (1.22–10.07)		2.05 (0.65–6.41)	
Per 1 pack-year of exposure		1.20 (1.03–1.39)	0.02	1.11 (0.95–1.29)	0.19
Pack-years of exposure to smoking by all family members					
0	44/187	1.0		1.0	
≤ 5	42/125	1.67 (1.01–2.75)		1.59 (0.92–2.72)	
> 5	40/91	2.55 (1.49–4.35)		2.00 (1.12–3.58)	
Per 1 pack-year of exposure		1.12 (1.06–1.19)	< 0.001	1.09 (1.03–1.17)	0.004
Urinary cotinine level (median, μg/g Cr)					
Low (0.82)	28/133	1.0		1.0	
Middle (1.44)	38/133	1.50 (0.86–2.63)		1.85 (1.01–3.40)	
High (3.25)	58/133	2.90 (1.69–4.98)		3.10 (1.71–5.63)	
			0.002		0.012

*OR* odds ratio*, CI* confidence interval, *Cr* creatinine

^a^Adjusted for child’s sex, age, indicator for preschools, mother’s age at delivery, maternal education, frequency of eating snacks, frequency of tooth brushing, feeding method until 3 months (breast, mixed, or bottle)

Table 3 shows the association of exposure to environmental tobacco smoke with the presence of decayed teeth. These associations were somewhat attenuated as compared to those with the presence of dental caries (decayed and/or filled teeth). However, the dose response relationships for pack-years of smoking by mother and all family members with presence of decayed teeth remained significant.

**Table 3:** OR and 95% CI for the presence of decayed teeth according to environmental tobacco smoke exposure at home

	*N* with caries/total	Crude OR (95% CI)	*P* trend	Multivariate OR (95% CI)^a^	*P* trend
Exposure to maternal smoking					
Never	86/356	1.0		1.0	
Past	2/5	2.09 (0.34–12.73)		2.24 (0.33–15.42)	
Current	17/44	1.98 (1.03–3.80)		1.89 (0.94–3.80)	
Pack-years of exposure to maternal smoking					
0	86/356	1.0		1.0	
≤ 3	6/23	1.11 (0.42–2.90)		1.11 (0.40–3.08)	
> 3	13/26	3.14 (1.40–7.03)		2.92 (1.23–6.96)	
Per 1 pack-year of exposure		1.30 (1.08–1.55)	0.005	1.28 (1.05–1.56)	0.01
Exposure to paternal smoking					
Never	43/206	1.0		1.0	
Past	10/27	2.23 (0.95–5.22)		2.06 (0.84–5.01)	
Current	52/172	1.64 (1.03–2.62)		1.44 (0.88–2.37)	
Pack-years of exposure to paternal smoking					
0	43/206	1.0		1.0	
≤ 4	32/93	1.99 (1.15–3.43)		1.79 (1.01–3.19)	
> 4	30/106	1.50 (0.87–2.57)		1.31 (0.74–2.31)	
Per 1 pack-year of exposure		1.08 (1.00–1.18)	0.06	1.05 (0.96–1.15)	0.28
Pack-years of exposure to smoking by other family members					
0	92/372	1.0		1.0	
≤ 4	5/17	1.26 (0.43–3.67)		1.14 (0.36–3.59)	
> 4	8/15	3.45 (1.22–9.78)		2.28 (0.75–6.91)	
Per 1 pack-year of exposure		1.19 (1.03–1.37)	0.02	1.12 (0.96–1.31)	0.14
Pack-years of exposure to smoking by all family members					
0	37/187	1.0		1.0	
≤ 5	37/125	1.72 (1.02–2.92)		1.61 (0.92–2.82)	
> 5	31/91	2.10 (1.19–3.68)		1.75 (0.96–3.20)	
Per 1 pack-year of exposure		1.09 (1.03–1.16)	0.002	1.07 (1.01–1.14)	0.03
Urinary cotinine level (median, μg/g Cr)					
Low (0.82)	26/133	1.0		1.0	
Middle (1.44)	34/133	1.41 (0.79–2.52)		1.66 (0.90–3.06)	
High (3.25)	43/133	1.97 (1.12–3.45)		2.02 (1.10–3.70)	
			0.04		0.10

^a^Adjusted for child’s sex, age, indicator for preschools, mother’s age at delivery, maternal education, frequency of eating snacks, frequency of tooth brushing, feeding method until 3 months (breast, mixed, or bottle)

Stratified analyses according to children’s age (3- to 4-year-old [*n* = 196] and 5- to 6-year-old [*n* = 209]) revealed a stronger association of exposure to maternal smoking with the presence of decayed caries in the latter group; the ORs for pack-years of smoking to maternal smoking were 2.17 (95% CI = 0.48–9.77, *P* for trend = 0.52) in 3- to 4-year-old children and 4.05 (95% CI = 1.26–13.01, *P* for trend = 0.01) in 5- to 6-year-old children. However, ORs for pack-years of exposure to smoking by all family members did not differ greatly; OR = 2.25, 95% CI = 0.73–6.98, *P* for trend = 0.09 in 3- to 4-years-old children and OR = 1.79, 95% CI = 0.83–3.85, *P* for trend = 0.15 in 5- to 6-year-old children. OR for the highest vs. lowest tertile of urinary cotinine level was greater in younger children group; OR = 4.67, 95% CI = 1.46–15.00, *P* for trend = 0.08 in 3- to 4-year-old children and OR = 1.50, 95% CI = 0.68–3.32, *P* for trend = 0.46 in 5- to 6-year-old children.

## Discussion

We found that exposure to maternal and paternal smoking was significantly associated with dental caries. We also observed significant dose relationships of the pack-years of exposure to smoking by the mother and all family members with the presence of dental caries. These data suggest that cumulative exposure to environmental tobacco smoke at home may affect the development of dental caries. So far, only two studies [5, 15] have assessed the pack-months of smoking by family members with regard to dental caries, and they did not do so separately for the mother, father, and other family members. Both studies observed a significant dose-response relationship similar to that in our study. Our results on maternal smoking status are also consistent with the findings of previous studies for early childhood [7, 8, 13, 16].

The association of dental caries with smoking by the father or other family members appeared to be weaker than that with maternal smoking, and the dose-relationship for pack-years of exposure to smoking by them was not statistically significant. In Japan, as younger children at preschool age generally spend a lot of time with their mothers at home, exposure to smoking by the mother assessed in terms of pack-years may have more effect than exposure to smoking by the father or other family members [4, 13]. In our study, the association for total pack-years of exposure to smoking by all family members should mainly reflect that for exposure to maternal smoking. Most previous studies assessed only the status of maternal smoking or household smoking as a whole in relation to dental caries. Even for the status of paternal smoking, only a few studies included this variable [4, 7, 13]. One study observed that paternal smoking status was significantly associated with the presence of dental caries [4], but the other two studies did not [7, 13].

We found that the urinary cotinine level was significantly associated with the presence of dental caries, and the dose-response relationship was significantly positive. A previous study evaluated the association of serum cotinine levels with the existence of unfilled and filled caries, and a significant dose-response relationship was observed for unfilled caries [10]. However, the OR did not monotonously increase with the higher cotinine level, and the authors suggested the possibility that there is a threshold of exposure above which children’s risk for caries does not continue to increase. This may be partially attributed to the fact that the cotinine level indicates the amount of nicotine exposure over a short period (about 3 days) [18]. Nevertheless, we cannot deny that current nicotine exposure reflects cumulative exposure to smoking, because the pack-years of exposure to all family smoking was well correlated with the urinary cotinine levels in our study.

Experimental evidence supports a positive association between environmental tobacco smoking exposure and dental caries. *Streptococcus mutans* is one of the major cariogenic microorganism in the oral cavity [19]. Nicotine enhances *Streptococcus mutans* biofilm formation and biofilm metabolic activity [20]. Nicotine also increases extracellular polysaccharides, which can attract other microorganisms, such as *Candida albicans*, onto the dental plaque [21]. In fact, in vivo, the caries-affected area on the molars was more expanded in cigarette smoke-exposed rats than in control rats [22]. In humans, tobacco smoking was associated with elevated levels of *Streptococcus mutans* and *Lactobacilli* [23, 24]. It is also possible that the immunosuppressive properties of smoking may have effects on the development of caries, as smokers had decreased levels of sIgA that was associated with the prevalence of dental caries [25].

Several limitations of the present study should be considered. Because of the cross-sectional study design, we cannot determine the cause–effect relationship. Another limitation was that the sample size was not sufficient enough to conduct meaningful analyses separately for decayed and filled teeth and for 3- to 4-year-old and 5- to 6-year-old children. Filled status could be determined not only by formation of dental caries but also by the extent of dental care that children received. In addition, although we asked mothers’ lifetime smoking history, we did not specifically ask whether they had smoked during pregnancy. Maternal smoking during pregnancy has been associated with dental caries among children in some studies [5, 6, 12, 26], but not all [27, 28]. Therefore, the observed positive association of maternal smoking with dental caries may be partially attributed to exposure to smoking during pregnancy. Because women who smoke during pregnancy are likely to continue smoking after delivery, it has been difficult to study the independent effects of in utero exposure to maternal smoking and postnatal exposure. However, Tanaka et al. [5] observed that both maternal smoking during pregnancy and postnatal household smoking were independently associated with an increased prevalence of dental caries. Since we observed a significant dose-response relationship for pack-years of smoking by the mother and by all family members, exposure to smoking after childbirth may have an effect on the development of dental caries regardless of in utero exposure. The misclassification of dental caries status is possible, because the filled status may be caused by cracked or broken teeth rather than decayed teeth. Previous studies have pointed out the possibility that smoking or the cotinine level is simply a marker for certain unmeasured true causes of caries formation [10, 11, 20]. Although we attempted to control for several potential confounders, we could not obtain information about the use of fluoride. However, few local governments in Japan fluoridate drinking water, which may have diluted a preventive effect of fluoride toothpaste. In addition, Tanaka et al. reported that the risk of dental caries with the use of fluoride among Japanese 3 years aged children was significantly positive (OR = 1.32) [5]. Three other studies among 3-year-old Japanese children did not observe an inverse association between the use of fluoride and dental caries [4, 13, 26]. Therefore, the observed associations between exposure to environmental tobacco smoke and caries experience are unlikely to be overestimates due to non-adjustment for fluoride use. Our study subjects were not representative of the preschool population in Japan, which may affect the generalizability of our findings. According to the national school health statistics for 2006, the percentages of 5-year-old children having dental caries (decayed and/or filled teeth) and decayed teeth were 55.2% and 33.5%, respectively [29]. Lower percentages were observed in our study (the corresponding values in our study subjects were 37.0% and 29.4%, respectively). In the National Health and Nutrition Survey 2006 [30], the percentages of never smokers among men and women aged 20–39 were 41.2% and 75.3%, respectively. Higher percentages of never smokers among parents in our study may partially explain the relatively low prevalence of dental caries among their children, but the evidence is equivocal. A somewhat lower prevalence of dental caries (43.7%) and decayed teeth (25.7%) was reported for 5-year-old children in Aichi Prefecture [29].

## Conclusion

In summary, we found that pack-years of exposure to smoking by the mother and by all family members, as well as urinary cotinine level, was associated with dental caries in preschool Japanese children. These data suggest that exposure to tobacco smoke may have dose-dependently influenced the development of dental caries. However, prospective studies that include the measurement of varying exposure levels over time are needed to delineate the effect of cumulative exposure to environmental tobacco smoke on the risk of dental caries.

## Abbreviations

CI: Confidence interval
LC-MS/MS: Liquid chromatography–tandem mass spectrometry
LOD: Limit of detection
OR: Odds ratio
SPE: Solid-phase extraction
